# Modeling Alexander disease with patient iPSCs reveals cellular and molecular pathology of astrocytes

**DOI:** 10.1186/s40478-016-0337-0

**Published:** 2016-07-11

**Authors:** Takayuki Kondo, Misato Funayama, Michiyo Miyake, Kayoko Tsukita, Takumi Era, Hitoshi Osaka, Takashi Ayaki, Ryosuke Takahashi, Haruhisa Inoue

**Affiliations:** Center for iPS Cell Research and Application (CiRA), Kyoto University, 53 Kawahara-cho, Shogoin, Sakyo-ku, Kyoto, 606-8507 Japan; Department of Cell Modulation, Institute of Molecular Embryology and Genetics (iMEG), Kumamoto University, 2-2-1 Honjo, Tyuou-ku, Kumamoto, 860-0811 Japan; Department of Pediatrics, Jichi Medical School, 3311-1 Yakushiji, Shimotsuke-shi, Tochigi 329-0498 Japan; Department of Neurology, Graduate School of Medicine, Kyoto University, 54 Kawahara-cho, Shogoin, Sakyo-ku, Kyoto, 606-8507 Japan

**Keywords:** Alexander disease (AxD), Glial fibrillary acidic protein (GFAP), Induced pluripotent stem cells (iPSCs), Disease modeling, Astrocytes, Rosenthal fibers, Heat-shock protein, Alpha-crystallin, Cytokine, Inflammatory response, Inherited astrocytopathy

## Abstract

Alexander disease is a fatal neurological illness characterized by white-matter degeneration and formation of Rosenthal fibers, which contain glial fibrillary acidic protein as astrocytic inclusion. Alexander disease is mainly caused by a gene mutation encoding glial fibrillary acidic protein, although the underlying pathomechanism remains unclear. We established induced pluripotent stem cells from Alexander disease patients, and differentiated induced pluripotent stem cells into astrocytes. Alexander disease patient astrocytes exhibited Rosenthal fiber-like structures, a key Alexander disease pathology, and increased inflammatory cytokine release compared to healthy control. These results suggested that Alexander disease astrocytes contribute to leukodystrophy and a variety of symptoms as an inflammatory source in the Alexander disease patient brain. Astrocytes, differentiated from induced pluripotent stem cells of Alexander disease, could be a cellular model for future translational medicine.

**Electronic supplementary material:**

The online version of this article (doi:10.1186/s40478-016-0337-0) contains supplementary material, which is available to authorized users.

## Introduction

Alexander disease (AxD) was first described by W. S. Alexander [1]. The clinical phenotypes of AxD are macrocephaly, frontal leukodystrophy and a variety of developmental delays with epileptic seizures, dysphagia, or bulbar/pseudobulbar signs. However, the severity of these clinical features differs among patients, being mostly dependent on the age of onset [2].

The common neuropathological feature of AxD is the presence of Rosenthal fibers, a unique cytoplasmic inclusion within astrocytes. Rosenthal fibers contain glial fibrillary acidic protein (GFAP), major astrocytic intermediate filament protein and molecular chaperones, including alpha-B-crystallin and other heat shock proteins [3, 4]. After extensive neuropathological investigations, missense mutations in GFAP have been identified as a genetic basis for AxD [5]. The discovery of the GFAP mutations opened the way to the development of model systems using tissue culture cells and transgenic mice for the study of AxD. Transgenic models recapitulated GFAP aggregations. However, it remained unclear how AxD mutations lead to protein aggregation in patient astrocytes as well as how mutant GFAP-expressing astrocytes contribute to neuronal degeneration [6].

In 2007, the discovery of a combination of transcription factors that could reprogram somatic cells into cells exhibiting pluripotency, called induced pluripotent stem cells (iPSCs), has provided researchers with a revolutionary tool to study human biology and diseases [7]. iPSCs can be derived from many somatic cell types, including easily accessible dermal fibroblasts and peripheral blood mononuclear cells [8, 9]. Similar to human embryonic stem cells (hESCs), iPSCs can self-renew and expand indefinitely in culture [7]. More importantly, they share the capacity to generate any cell types in the body, a property that is particularly useful for the study of neurological diseases. The pluripotency of iPSCs enables the production of astrocytes for disease modeling [10–12]. This remarkable feature of iPSCs facilitates the study of brain cell types that are difficult to obtain from living individuals, including human astrocytes. To directly examine AxD patient astrocytes, we established iPSCs from three AxD patients, with different *GFAP* mutations, respectively, and three healthy individuals without *GFAP* mutation. In a recent review of more than 215 cases of AxD with *GFAP* mutation, AxD was divided into 2 groups: type I was characterized by early onset, seizures, megalencephaly, and typical leukodystrophy as MRI features, and type II with a later age at onset characterized by brainstem features and atypical MRI findings [13]. Two patients in this study showed type I clinical phenotype and one patient showed type II clinical phenotype. In this study, AxD iPSC-derived astrocytes showed GFAP-positive aggregates, like Rosenthal fibers, and also exhibited altered cytokine release. The strategy of this study was to provide an effective and versatile way of pathogenic investigation and drug screening for AxD and other astrocyte-relevant diseases.

## Materials and methods

### Human subjects

Skin or blood samples were obtained from healthy controls or patients with Alexander disease. The study was approved by the Institutional Review Board and Ethics Committees of the University of Kyoto and Kumamoto University and written informed consent was obtained from all participants in this study.

### Generation of human iPSCs

In this application study, we used dermal fibroblasts or blood cells as patient somatic cells to prepare iPSCs [8, 12, 14]. For the iPSC clones of HC1, HC2, HC3, Alex1, and Alex3, episomal vectors were used to introduce a reprogramming factor (SOX2, KLF4, OCT4, L-MYC, LIN28, siRNA for p53) to the somatic cells, which were seeded onto SNL feeder cells. The next day, the medium was changed from a dermal fibroblast medium to a human ES cell medium (ReproCell, Yokohama, Japan) comprising 4 ng/mL of bFGF (Wako Chemicals, Osaka, Japan); the medium was replaced every other day, and after 30 days, about 20 iPSC colonies were picked up. Later, the presence or absence of residual plasmid was confirmed by PCR, and clones without residual plasmid were selected. Selected clones were run through karyotype analysis, and normal karyotype clones were analyzed. For the iPSC clones of Alex2, human iPSCs were generated by using Sendai virus vector as described previously [14].

### *In vitro* differentiation into three germ layers

CTK was used to harvest the iPSCs, and an embryoid body (EB) was formed [12]. Cell masses were cultured in DMEM/F12 (Thermo Fisher Scientific, Waltham, MA) comprising 20 % knockout serum replacement (KSR, Thermo Fisher Scientific), 2 mM L-glutamine (Thermo Fisher Scientific), 0.1 M nonessential amino acids (NEAA, Thermo Fisher Scientific), 0.1 M 2-mercaptoethanol (Thermo Fisher Scientific), and 0.5 % penicillin/streptomycin. The medium was replaced every other day, and the EB after 8 days was cultured for another 8 days in DMEM comprising 10 % FBS on a gelatin-coated coverslip.

### Differentiation and enrichment of astrocytes

Human iPSCs were dissociated to single cells and quickly reaggregated in U-bottom 96-well plates for suspension culture (Greiner Bio-One, Frickenhausen, Germany), pre-coated with 2 % Pluronic F-127 (Sigma-Aldrich, St. Louis, MO) in 100 % ethanol. Cell aggregates, called embryoid bodies (EBs), were cultured in ‘DFK5% medium’ (DFK5%; DMEM/F12 (Thermo Fisher Scientific) supplemented with 5 % v/v KSR, 1x NEAA, 1x Glutamax (Thermo Fisher Scientific), 0.1 M 2-mercaptoethanol (Thermo Fisher Scientific)) with 2 μM dorsomorphin (Sigma-Aldrich) and 10 μM SB431542 (Cayman Chemical, Ann Arbor, MI) in a neural inductive stage (day 0 to 8). After neural induction, EBs were transferred onto Matrigel (Corning, Tewksbury, MA)-coated 6-well culture plates and cultured in DFK5% supplemented with 1x N2 supplement (Thermo Fisher Scientific) and 2 μM dorsomorphin in the patterning stage (day 8 to 24). A large number of neural stem cells (NESTIN-positive) were observed to migrate from the EB core. After the patterning stage, migrated neural stem cells were separated from the plate bottom using Accutase (Innovative Cell Technologies, Inc., San Diego, CA) and cultured in Neurobasal medium FULL, Neurobasal Medium (Thermo Fisher Scientific) supplemented with 1x N2 supplement, 1x Glutamax, 10 ng/ml BDNF (Peprotech, Rocky Hill, NJ), 10 ng/ml GDNF (Peprotech) and 10 ng/ml NT-3 (Peprotech) on Matrigel-coated 6-well culture plates or cover-slips (day 24 to 60). At day 60, iPS-derived neural cells were plated at 400,000-2,000,000 cells per 90-mm dish without any coating in DMEM/F12 Glutamax (Thermo Fisher Scientific) supplemented with 1x N2 supplement, 10 ng/ml EGF (Peprotech), 12 ng/ml basic FGF (Peprotech) and 2 μg/ml heparin (Nacalai Tesque, Kyoto, Japan). After passage, neurons could not attach to a non-coated polystyrene dish surface or they died by anoikis. On the other hand, astrocytes and a limited number of oligodendrocyte precusors could attach and proliferate. By repeated passage in the same manner at days 90, 120, 150 and 180, astrocytes increased their own abundance ratio and showed positive GFAP immunostaining.

### Immunofluorescent study

The iPSCs or differentiated astrocytes were immobilized at room temperature for 30 min in 4 % paraformaldehyde (pH 7.4), and washed with PBS. The cells were then permeabilized for 10 min with PBS comprising 0.2 % Triton X-100. A non-specific reaction was also run for 60 min at room temperature in PBS comprising 10 % donkey serum; primary antibodies were reacted overnight, and fluorescently labeled secondary antibodies were reacted and observed. DAPI (Thermo Fisher Scientific) was used for nuclear staining. The following primary antibodies were used: NANOG (R&D Systems, Minneapolis, MN, 1:50), TRA1-60 (Millipore, Darmstadt, Germany, 1:1,000), SOX-17 (R&D Systems, 1:50), αSMA (Dako, Glostrup, Denmark, 1:3,000), Tuj1 (Covance, 1:3,000), S100β (Abcam, Cambridge, UK, 1:400), GFAP (DAKO, 1:2,000 or Santa Cruz Biotechnology, Dallas, TX, 1:400), alpha-B crystallin (Millipore, 1:400), and N-cadherin (Santa Cruz Biotechnology, 1:50). Rhodamine phalloidin (Thermo Fisher Scientific, 1:1,000) were used for F-actin staining. Immunostained cells were analyzed using In Cell Analyzer 6000 (GE Healthcare, Chicago, IL) or the super-resolution structured illumination microscopy with 100 x objective lens (N-SIM system, Nikon Instruments, Tokyo, Japan).

### Transmission electron microscopy

Briefly, iPSC-derived astrocytes were cultured on plastic coverslip (CellDesk, Sumitomo Bakelite Co., Ltd., Tokyo, Japan) and fixed in 4 % paraformaldehyde/2 % glutaraldehyde/0.1 M phosphate buffer at 4 °C, washed in isotonic phosphate-buffered sucrose, and then post-fixed in 1 % osmic acid. Specimens were dehydrated with ethanol and propylene oxide and subsequently embedded in epoxy resin. Ultrathin sections were cut with an ultramicrotome, mounted on grids, stained with uranyl acetate and lead citrate, and examined by using a Hitachi H-7650 electron microscope (Hitachi, Tokyo, Japan).

### Microarray and pathway analysis for differentiated astrocytes

Total RNA from differentiated neural cells was extracted by RNeasy micro kit (QIAGEN, Hilden, Germany) and altered into ragmented/biotinylated cDNA by GeneChip® WT PLUS Reagent Kit (Affymetrix, Santa Clara, CA). Fragmented cDNA samples were hybridized with GeneChip Human Gene 2.0 ST Array (Affymetrix). Each sample was hybridized once with the one-color protocol. Arrays were scanned with a GeneChip® Scanner 3000 7Gt (Affymetrix). Data were analyzed by GeneSpring GX7.3.1 software (Agilent Technologies, Santa Clara, CA) to create the list of gene sets. The normalized data have been deposited at Gene Expression Omnibus (GEO, http://www.ncbi.nlm.nih.gov/geo/) with accession number GSE83374. For pathway analysis, we adopted gene sets for Ingenuity Pathway Analysis (IPA software, QIAGEN), and seek altered canonical pathways. To understand the upstream of pathway changes, the activation status of the functions/pathways was predicted using the IPA Upstream Regulator Analysis Tool, by calculating a regulation Z-score and an overlap p-value, which were based on the number of known target genes of interest pathways/functions, expression changes of these target genes and their agreement with literature findings. It was considered significantly activated (or inhibited) with an overlap p-value ≤ 0.05 and an IPA activation Z-score ≥ 1.2 (or ≤ −1.2). The detailed descriptions of IPA analysis are available under “Upstream Regulator Analysis”, “Biological Functions Analysis”, and “Ingenuity Canonical Pathways Analysis” on the IPA website (http://www.ingenuity.com).

#### Immunoblots

Cells were lysed in RIPA buffer (50 mM Tris-HCl buffer, pH 8.0, 150 mM NaCl, 1 % NP-40, 0.5 % deoxycholate, 0.1 % SDS, protease inhibitor cocktail (Roche Diagnostics, Basel, Switzerland), phosphatase inhibitor cocktail (Roche Diagnostics)). Each 10 μg sample of protein was subjected to SDS-PAGE (5-20 % gradient SDS-polyacrylamide gels, BIOCRAFT, Tokyo, Japan), and separated proteins were transferred to polyvinylidene fluoride membrane (HybondTM-P, GE Healthcare). The membranes were incubated with primary antibodies, followed by appropriate secondary antibodies, and then visualized using ECL prime (GE Healthcare). For dot-blot analysis, cell lysate samples (each 2 or 4 μg/spot) were loaded on a nitrocellulose membrane. The membranes were incubated with primary antibodies, followed by appropriate secondary antibodies, and then visualized using ECL prime (GE Healthcare). The images were acquired on LAS 4000 (GE Healthcare). The intensity of the protein band was analyzed using Fiji (http://fiji.sc/). The following primary antibodies were used: N-cadherin (1:1,000, Santa Cruz Biotechnology), GAPDH (1:3,000, Abcam), 4E-BP1 (1:1,000, Cell Signaling Technology (CST), Danvers, MA), Phospho-4E-BP1 (Ser65) (1:1,000, CST), eIF4B (1:1,000, CST), Phospho-eIF4B (Ser406) (1:1,000, CST), eIF4E (1:1,000, Abcam), and Phospho-eIF4E (Ser209) (1:1,000, Abcam).

### Electrochemiluminescence assays for cytokines

Differentiated astroglial cells were replated at 4 x10^4^ cells per well in 96-well plates coated with 0.1 % gelatin. Three days after replating, all culture medium was replaced with 100 μL of fresh astrocyte medium. To assess extracellular cytokine release, conditioned media were harvested for further analysis. As positive control of massive cytokines release, 1 μg/mL LPS was added to NC1 astrocytes. Cytokines in culture media were measured by human Cytokine Demonstration 10-Plex tissue Culture Kits (Meso Scale Discovery, Rockville, MD). This assay uses each antibody to capture each cytokine and SULFO-TAG-labeled different specific antibodies for detection by electrochemiluminescence with Sector® Imager 2400 (Meso Scale Discovery). Ten kinds of cytokines, including IL-1β, IL-2, IL4, IL-5, IL-6, IL-8, IL-10, IL-12p70, GM-CSF and TNFα, were assayed. The concentrations of IL-1β, IL4, IL-5, IL-6, GM-CSF and TNFα were quantified by using standard recombinant proteins, but the signals of IL-2, IL-8, IL-10 and IL-12p70 were not able to be detected in the dynamic range of the standard curve (0.05-10,000 pg/ml).

### Statistics

Comparisons of the mean among three groups or more were performed by one-way or two-way analysis of variance (ANOVA) followed by post-hoc test using Tukey–Kramer method (JMP software version 9.0, SAS Institute Inc., Cary, NC). *P* values < 0.05 were considered significant.

## Results

### Generation and characterization of AxD-specific iPSCs

**Fig. 1:**
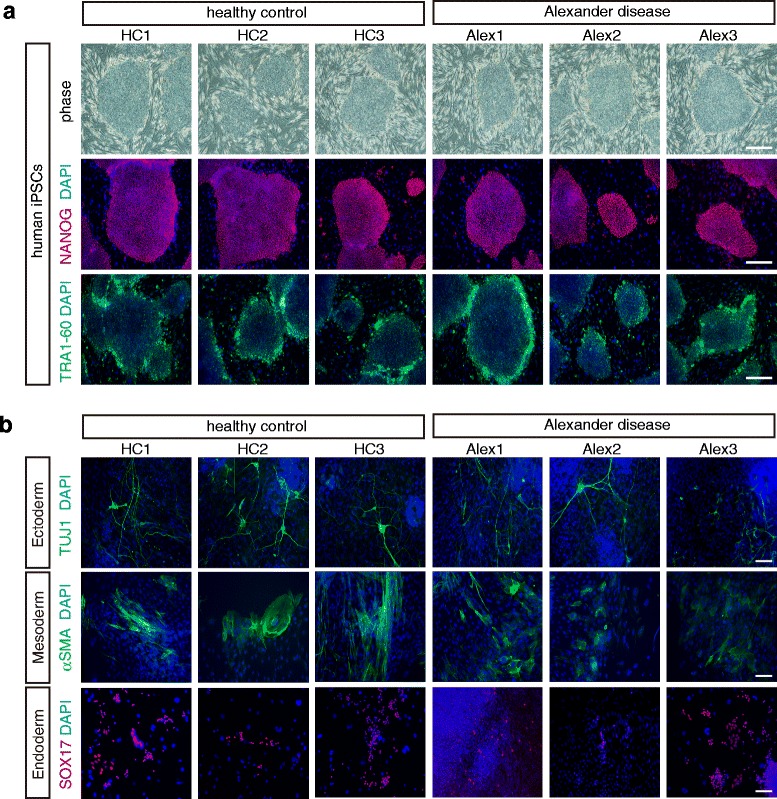
Generation of iPSCs from Alexander disease patients and healthy controls. **a** Morphology and expression of human embryonic stem cell markers. iPSCs from both controls and patients with Alexander disease showed ESC-like morphology (phase image) and expressed pluripotent stem cell markers, NANOG and TRA1-60. Scale bars = 200 μm. **b** *In vitro* differentiation of established iPSCs to representative three-germ layer: TUJ1 (ectoderm), αSMA (mesoderm), and SOX17 (endoderm). Scale bars = 50 μm

In the present study, we generated iPSCs from three AxD patients with heterozygous *GFAP* mutation (Alex1, Alex2 and Alex3) and three healthy controls (HC1, HC2, and HC3) (Table 1). The disease onset of Alex1 and Alex2 was infantile and that of Alex3 was adult (Table 1). Primary cultures of somatic cells from all six individuals were independently reprogrammed to iPSCs, as judged by colony morphology, similar to human embryonic stem cells (ESCs), growth dynamics, and sustained long-term passaging (>20 passages) (Fig. 1a). The established iPSCs expressed NANOG and TRA1-60, markers of pluripotency (Fig. 1a). The pluripotency of the iPSCs was also evaluated *in vitro* through the formation of EBs. All iPSC lines spontaneously differentiated into cell types of the three embryonic germ layers as indicated by expression of the specific markers, including TUJ1 (ectoderm marker), αSMA (mesoderm marker), and SOX17 (endoderm marker) (Fig. 1b).

**Table 1:** Summary of iPSCs in this study

clone name	clinical character	GFAP genotype	Sex	Age at onset	Age at sampling
HC1	healthy	wild	female	-	36
HC2	healthy	wild	female	-	67
HC3	healthy	wild	male	-	74
Alex1	Alexander disease type I	R239C (c. 729 C > T)	male	2	6
Alex2	Alexander disease type I	E63K (c.205 G > A)	female	3	10
Alex3	Alexander disease type II	R276L (c.827 G > T)	female	33	45

*Abbreviations*: *GFAP* Glial fibrillary acidic protein, *HC* Healthy control

Alex1 was generated from patient fibroblasts (GM16825) from Coriell Institute (Camden, NJ)

### Differentiation of iPSCs into astrocytes

**Fig. 2:**
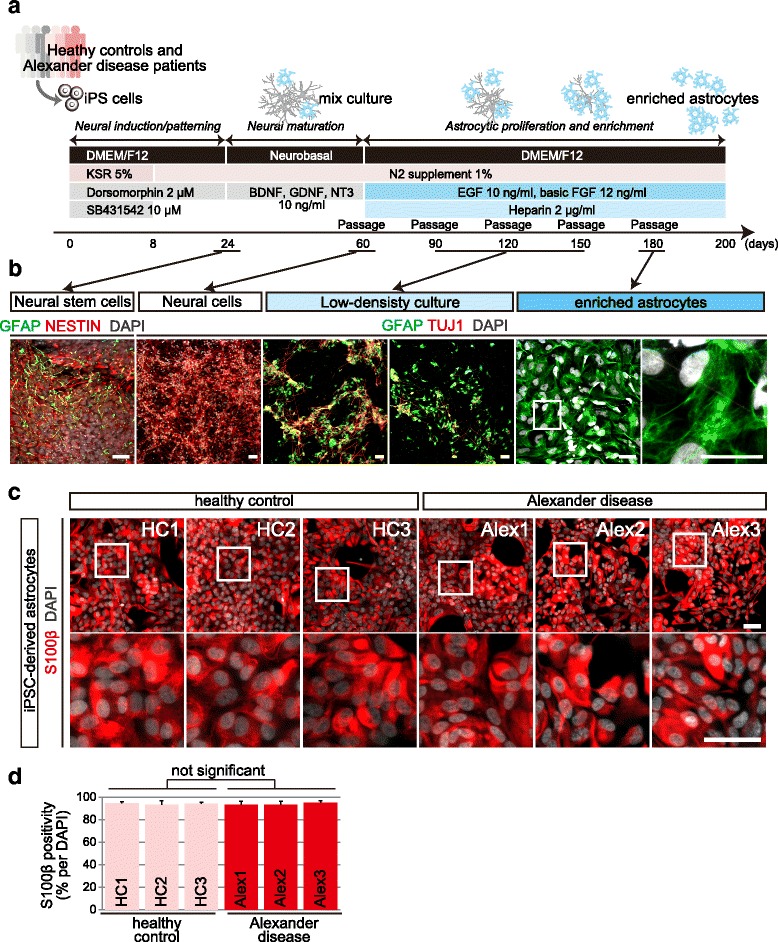
iPSCs from AxD patients and healthy controls could differentiate into astrocytes with high purity. **a** Schematic procedures for astroglial differentiation. **b** Differentiated iPSCs at day 24 expressed neural stem cell markers NESTIN and GFAP. Neural cells at day 60 expressed neuronal or astrocytic marker TUJ1 or GFAP. Most enriched astrocytes expressed GFAP. Scale bars = 20 μm. **c** Estimation of astroglial differentiation from control and AxD iPSCs. After 180 days of differentiation, astrocytes were immunostained with an antibody against S100β (red color). Scale bars = 20 μm. **d** Calculated purity of astrocytic differentiation Data represent mean ± SD (biological replicates, n = 3 from randomly picked fields per clone). Two-way analysis of variance (ANOVA) did not show significant variation. F (5, 12) =0.2432; p = 0.935

**Fig. 3:**
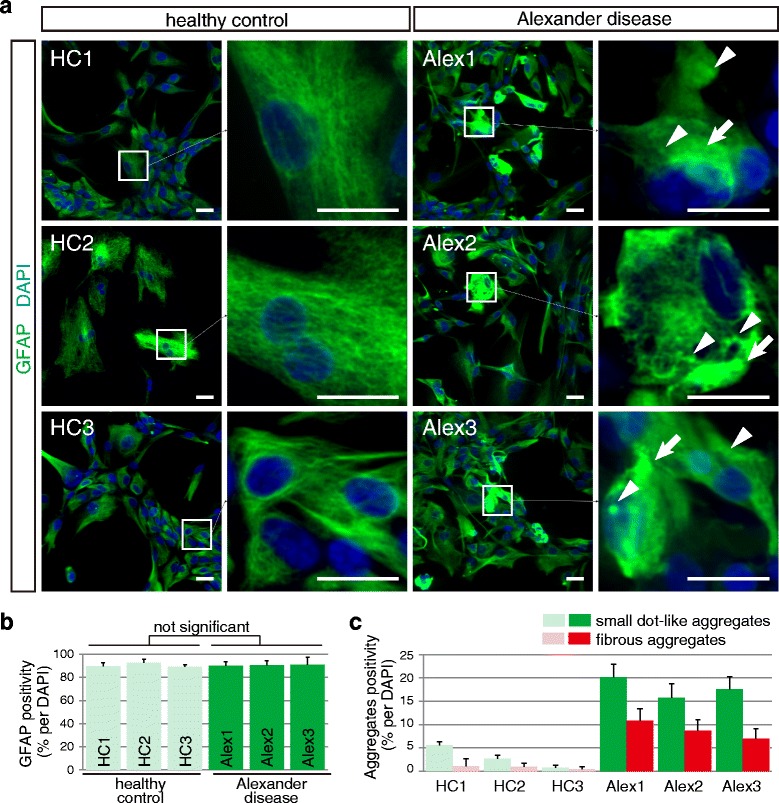
Astrocytes of Alexander disease showed GFAP-positive aggregates. **a** Most iPSC-derived astrocytes showed GFAP-positive staining (*green color*). In healthy control, GFAP showed filamentous structure. In Alexander disease, GFAP mainly showed filamentous structure, but also fibrous/amorphous (*arrows*) or dot-like (*arrowheads*) aggregates. Scale bars = 5 μm. **b** Calculated purity of astrocytic differentiation data represent mean ± SD (biological replicates, n = 3 from randomly picked fields per clone). **c** Calculated positivity of GFAP aggregates. Data represent mean ± SD (biological replicates, n = 3 from randomly picked fields per clone)

The astrocytic differentiation protocol for human iPSCs was modified from our previous method [12] (Fig. 2a). In the neural patterning stage, differentiated cells expressed NESTIN (marker of neural stem cells) or GFAP (marker of radial glia in cortical development) (Fig. 2b). After 2 months, differentiated cells abundantly expressed TUJ1 (neuronal marker) (Fig. 2b). By repeating low-density passage, differentiated neurons, without proliferation, failed to attach to the dish and were selectively removed. After five passages and more than 6 months of cultivation, iPSC-derived astrocytes were enriched (Fig. 2b). Differentiated astrocytes abundantly expressed S100β (Fig. 2c) and GFAP (Fig. 3a and b), which are commonly used as astrocytes markers. We did not observe any obvious difference in astrocytic differentiation efficacy among all individuals (Fig. 2d).

### GFAP aggregates in iPSCs-derived astrocytes from AxD

**Fig. 4:**
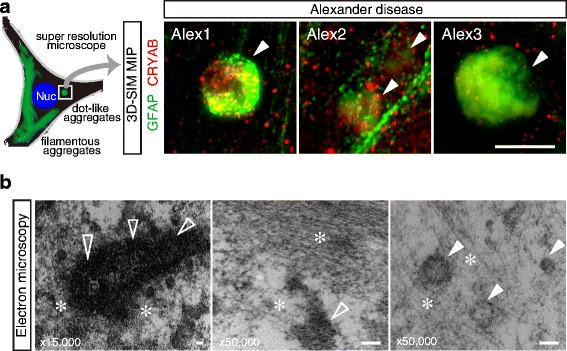
High resolution imaging of aggregates in AxD astrocytes with 3D-SIM or electron microscopy. **a** Super resolution imaging of GFAP aggregates with dot-like pattern showed accumulated particles of both GFAP and alpha-B crystalline (CRYAB). Scale bar = 200 nm. **b** Electron microscopy of AxD astrocytes. Electron-dense amorphous-appearing structures (*open arrow head*) or granular (*closed arrow head*) structures, surrounded by filamentous structure (*). Scale bars = 100 nm

To evaluate the *in vitro* recapitulation of Rosenthal fibers, we visualized GFAP of iPSC-derived astrocytes by immunofluorescent staining. Nearly all iPSC-derived astrocytes showed positive staining of GFAP (Fig. 3a and b). GFAP of healthy control astrocytes formed fine filaments distributed throughout the cytoplasm in a cytoskeletal array (Fig. 3a, panels of HC1, 2, and 3). In contrast, a proportion of GFAP in AxD astrocytes formed fibrous aggregates, similar to Rosenthal fibers of AxD brain, and also small dot-like patterns (Fig. 3a, panels of Alex 1, 2, and 3). These fibrous aggregates were formed in 5-10 % of AxD, and were rarely observed in healthy controls. Small dot-like aggregates were formed in 15-20 % of AxD and in a few of the healthy controls (Fig. 3c). To characterize small dot-like inclusions in detail, we visualized GFAP-positive dots using super-resolution structured illumination microscopy (N-SIM system). GFAP-positive dots, with a diameter of 50-200 nm, showed a cloud-like amorphous structure, adjacent to normal GFAP filament, and were co-immunostained with alpha-B crystallin particles (Fig. 4a). In addition to super-resolution microscopy, we observed cytosolic aggregates in AxD astrocytes by using electron microscopy. In AxD astrocytes, electron-attenuated, granular, or amorphous-appearing structures, surrounded by filamentous structure, were observed and determined as Rosenthal fiber-like structures (Fig. 4b). Overexpression of GFAP might contribute to astrocyte dysfunction in AxD as was shown in initial studies of overexpressing wild type GFAP in transgenic mice, which resulted in the formation of Rosenthal fibers indistinguishable from those found in Alexander disease patients. To investigate the GFAP dose effects on aggregates formation, we quantified the GFAP expression and found increased GFAP in AxD astrocytes (Additional file 1: Figure S1).

### Pathway analysis of alteration in global gene expression patterns

**Fig. 5:**
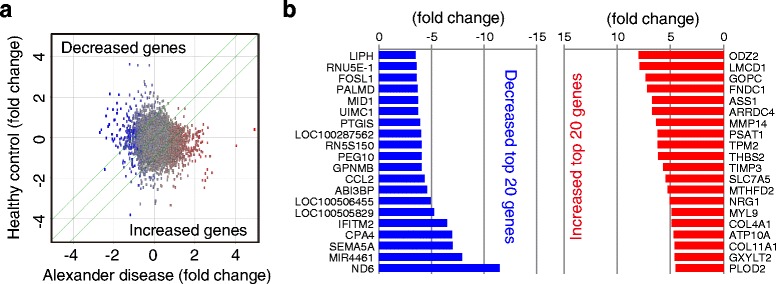
Gene expression comparison between healthy control and Alexander’s disease astrocytes. **a** Scatter plot showing the 2-fold upregulated and downregulated genes (red and blue dots, respectively) in the astrocytes of Alexander disease. **b** List of increased and decreased top-20 genes (red and blue columns, respectively)

To uncover molecules involved in the AxD astrocyte pathogenesis, we analyzed global gene expression profiles of iPSC-derived astrocytes (Fig. 5a and b). Among 40,716 probe sets, we created a gene set with altered expression in AxD astrocytes versus control astrocytes (fold-change ≥ 2 fold). By adapting this gene set to the pathway analysis software, we investigated the background pathway of the AxD pathomechanism. Pathway analysis revealed altered function of cellular adherence (Additional file 2: Figure S2) and transcription change via mTORC1/mTORC2 (Additional file 3: Figure S3).

**Fig. 6:**
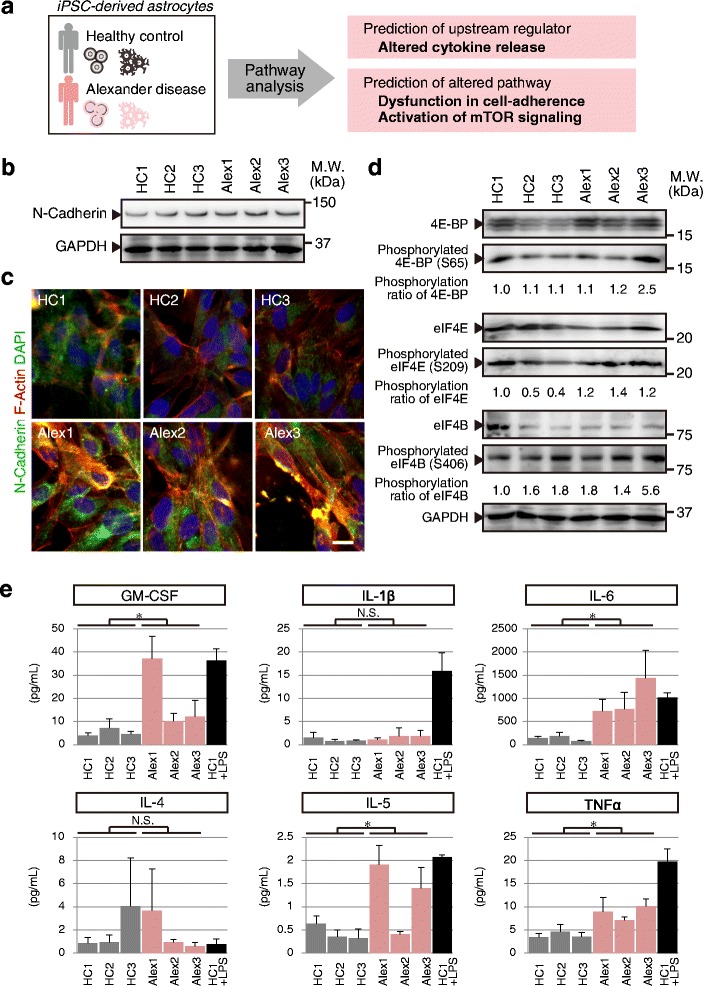
Pathway analysis revealed altered status of cell-adhesion, mTOR, and cytokine release in Alexander disease astrocytes. **a** Schema of pathway analysis and upstream prediction analysis. **b** Whole-cell lysates of iPSC-derived astrocytes were prepared and equivalent amounts of total protein were loaded per lane on a polyacrylamide gel for Western blot (WB) analysis using N-cadherin or GAPDH antibodies shown on the left. **c** Astrocytes were immunostained with an antibody against N-cadherin (*green color*) and F-actin (*red color*) was also visualized by using Rhodamine phalloidin. Scale bar = 10 μm. **d** Whole-cell lysates were prepared and equivalent amounts of total protein were loaded per lane on a polyacrylamide gel for WB analysis using the panel of antibodies shown on the left. The values shown below each blot represent the ratio of phosphorylated/total band densities, calculated and normalized to the ratio in “HC1” astrocytes. **e** Quantification of cytokine release from iPSC-derived astrocytes. Gray/pink-colored columns indicate astrocytes of healthy controls/Alexander disease. Black-colored column indicates healthy astrocytes with addition of LPS as positive control of cytokine release. LPS: lipopolysaccharide. (*, p < 0.05, N.S.: not significant) Data represent mean ± SD (biological replicates, n = 3)

From the results of cellular adherence pathway analysis, the expression of cell adhesion molecules (CAM), including the cadherin family, was altered in AxD astrocytes. In iPSC-derived astrocytes of AxD, gene expression and protein level of N-cadherin were increased, and those of E-cadherin were decreased (Additional file 2: Figure S2 and Fig. 6b). Cadherin is known to play an important role in the interactions between cells or their surrounding matrix, and also to affect the cell morphology [15]. The majority of iPSC-derived astrocytes showed polygonal shape, and less than 20 % showed stellate or star-like shape. However, we could not find any distinct difference in cell shape between control and AxD astrocytes. To investigate the detailed structural changes in AxD astrocytes, we performed an immunfluorescence study of N-cadherin and F-actin. The signal intensity of N-cadherin was increased in AxD astrocytes, but the distribution of N-cadherin or F-actin was similar between control and AxD (Fig. 6c).

Additionally, the altered pattern of global gene expression in AxD astrocytes suggested activation of the mTOR pathway. Active mTOR promotes protein synthesis by phosphorylating 4E-BPs on several sites that relieve their binding to eIF4E, eIF4G or eIF4B. eIF4E mediates binding of eIF4 large protein complex to the 5’ cap structure of mRNAs. On the other hand, 4E-BPs in their hypophosphorylated state bind to eIF4E competitively, inhibiting the association of eIF4E and eIF4G and leading to a block in translation [16]. Thus, we evaluated mTOR activation by western blotting with antibodies, specific to phosphorylated 4E-BP, eIF4E, eIF4G, and eIF4B (Fig. 6d). As well as the prediction of pathway analysis, the phosphorylation statuses of 4E-BP, eIF4E, eIF4G, and eiF4B were upregulated in AxD astrocytes (Fig. 6d), indicating activated mTOR pathway.

To understand the upstream of these two pathway changes, the activation status of the functions/pathways was predicted. We can predict that altered gene expression and pathways in AxD is regulated by inflammatory cytokines (Fig. 5c). To test the status of cytokines from iPSC-derived astrocytes, cytokines secreted in culture medium were quantified (Fig. 5d). The secretion of GM-CSF, IL6, IL5 and TNFα was significantly increased in AxD astrocytes (Fig. 5d). In contrast, the secretion of IL1β and IL4 was not altered. Additionally, immunostaining study showed intracellular dots of each cytokine in iPSC-derived astrocytes (Additional file 4: Figure S4). These results indicated that AxD astrocytes secrete more inflammatory cytokines and affect the neural circumstances of AxD patient brain.

## Discussion

We generated iPSCs from AxD patients and differentiated them into astrocytes exhibiting GFAP-positive aggregates in cytosol. A part of the cytosolic aggregates was fibrous and had high immunoreactivity to GFAP antibody, resembling Rosenthal fibers of AxD brain. On the other hand, the filamentous structure of GFAP, which is the major intermediate filament of astrocytes, was almost absent in astrocytes with fibrous aggregates. In spite of this, however, we could not find any distinct morphological alteration in the shape of astrocytes with fibrous aggregates. We speculate that other species of intermediate filament proteins, including vimentin, may have compensated for the diminished GFAP-filament to maintain the cellular shape [17]. In addition to the fibrous aggregates, AxD astrocytes displayed small, round shape of GFAP-positive aggregates, described as a “small dot-like pattern” in the results. These small dots were similar to those of previous reports using cancer cell line models with overexpression of GFAP (overexpression models: OE models) [5, 18, 19]. These small dots in OE models were observed as irregular dots with or without sand-like diffuse staining patterns. The OE models with mutant GFAP-aggregates did not show any normal filamentous pattern of GFAP. However, anti-GFAP staining of iPSC-derived astrocytes of AxD showed both normal filamentous structure and small-dot aggregates in the same cells. Furthermore, the frequency of the appearance of small-dot aggregates was greater than that of fibrous aggregates. We speculate that small-dot aggregates, co-existing with wild filamentous GFAP, are a premature form of fibrous aggregates and transformed into fibrous aggregates after a period of time and/or accumulation of cellular stress. We also could observe electron-dense amorphous-appearing structures or granular structures, surrounded by filamentous cytoskeleton. These structures are similar to Rosenthal fiber in AxD patients brain, but electron-density of structures in iPSC-derived astrocytes were not as high as that of AxD brain. Relatively low electron-density might reflect the early stage of Rosenthal fiber, which can be an advantage in drug development to modify early pathomechanisms of AxD.

Secondly, we investigated how GFAP aggregates elicit the neurodegenerative process. By comparing global-gene expression between control and AxD astrocytes, we focused on cell-adhesion pathway and mTOR pathway. GFAP is an important cytoskeleton protein, but cell shape and cell proliferation are similar between control and AxD astrocytes. In our study, the iPSC-derived astrocytes were cultivated in the absence of neurons or other extracellular matrix and might require years of observation after transplantation into in vivo brain, to recapitulate morphological phenotypes, such as the increased ratio of reactive astrocytes. However, N-cadherin protein was increased in AxD astrocytes. Among the cadherin family, N-cadherin has been classified as “nerve-derived”, and is known as a key CAM in the brain. N-cadherin has also been reported to be upregulated via cellular-stress signaling after brain injury by using the N-cadherin knockout model [20]. We speculated that GFAP aggregates in AxD astrocytes can evoke cellular stress and upregulated N-cadherin as a stress response. In the case of in vivo, altered cell-adhesion via N-cadherin also might affect the cell-to-cell interaction among neurons, oligodendrocytes, microglia and astrocytes, and consequently could lead to clinical phenotypes of AxD.

Furthermore, we also focused on activation of the mTOR pathway. Activation of the mTOR cascade is known as a characteristic feature of the initial stress response, and is related to reactive astrocytes in brain pathologies [21]. According to a previous study by using GFAP Tg; Gfap+/R236H model mice, the activation through the mTOR pathway appears to be an early change, while the later, more severe pathology is accompanied by mTOR inactivation [22]. Considering these pieces of evidence, our iPSC-derived astrocytes reflect the early phase of the AxD pathomechanism, and should be applicable to the development of drugs for an initial insult of GFAP aggregates.

Previous neuropathological investigations have described that lymphocytic infiltration or microglial activation was not massive in AxD brain [2, 23]. Olanbarria *et al*. investigated detailed alteration of the cytokine network in AxD model mice with both OE of human wild-type *GFAP* and heterozygous knock-in of mice *Gfap* R236H, and detected inflammatory response [24]. Consistent with this report, in our study, AxD astrocytes from patient iPSCs exhibited increased amounts of secreted GM-CSF, IL5, IL6, and TNFα. GM-CSF, IL6, and TNFα are well known as proinflammatory cytokines and are upregulated in various kinds of white matter diseases, including multiple sclerosis (MS) and neuromyelitis optica (NMO) [25–28]. IL-5 is a Th2 cell-type cytokine that is secreted by astrocytes and microglia [29], turning on the switch of inflammatory response by activating microglia to upregulate inflammatory response in the brain, cooperating with GM-CSF [30, 31]. IL-5 is also upregulated not only in inflammatory conditions with parasitic infections but also in neurodegenerative disorders, including Parkinson’s disease [32]. In addition, clinically, AxD, especially type 2, shows step-wise or stroke-like progression, which is similar to the typical progression of other white matter diseases involving oligodendrocytes, MS and NMO. These findings suggest that the neuroinflammatory process promoted by proinflammatory astrocytes may be involved in the pathogenesis of AxD, and that immunomodulation approaches [33] targeting astrocytopathy would be a candidate therapy for AxD. Recent studies showed that astrocytes themselves can secrete cytokines and have responsibility for extrinsic factors, including LPS [34–36]. The cell population of iPSC-derived astrocytes did not show microglial markers. So, astrocyte-derived cytokines and chemokines might play both neuroprotective and neurotoxic roles in AxD, and could be a phenotypic target of future drug development by the use of an iPSC-derived astrocyte platform.

## Conclusions

iPSCs from AxD patients were used to clarify disease phenotypes of astrocytes, which are the target cells of AxD. iPSC-derived astrocytes from AxD patients showed GFAP-aggregates resembling Rothental fibers and altered release of cytokines such as in white matter disease. Patient-specific iPSCs of AxD would provide a feasible platform for the study of inherited astrocytopathies, and further studies focusing on pathological crosstalk between astrocytes and other types of cells in the brain might lead to novel therapies for AxD.

## Ethics approval and consent to participate

The study was approved by the Institutional Review Board and Ethics Committees at the University of Kyoto and Kumamoto University, and written informed consent was obtained from all participants in this study.

## Additional files

Additional file 1: Figure S1. GFAP expression of iPSC-derived astrocytes. Gene expression of GFAP was quantitatively analyzed with RT-qPCR. Two-way analysis of variance (ANOVA) showed significant variation. F (5, 12) =9.2490; p = 0.0008. Post hoc analysis revealed significant increases in GFAP expression in Alex1 and Alex3 (*, p < 0.05). Data represent mean ± SD (biological replicates, n = 3). (PDF 343 kb) Additional file 2: Figure S2. Alteration in cellular adherence pathway. Gene expression changes were described within epithelial adherens junction signaling. Red to orange color = increased in Alexander disease. Blue to green color = decreased in Alexander disease, Grey color = not altered. (PDF 3.50 MB) Additional file 3: Figure S3. Alteration in mTORC1/mTORC2 pathway. Gene expression changes were described within mTOR signaling. Red to orange color = increased in Alexander disease. Blue to green color = decreased in Alexander disease, Grey color = not altered. (PDF 3.63 MB) Additional file 4: Figure S4. Immunofluorescent study of cytokines in iPSC-derived astrocytes. iPSC-derived astrocytes showed positive staining of IL-1β, IL-6, IL-5 (green color) and , GM-CSF, TNFα, IL-4 (red color). Scale bar = 5 μm. (PDF 3.32 MB)
