# Erratum to: Modeling Alexander disease with patient iPSCs reveals cellular and molecular pathology of astrocytes

**DOI:** 10.1186/s40478-016-0366-8

**Published:** 2016-09-16

**Authors:** Takayuki Kondo, Misato Funayama, Michiyo Miyake, Kayoko Tsukita, Takumi Era, Hitoshi Osaka, Takashi Ayaki, Ryosuke Takahashi, Haruhisa Inoue

**Affiliations:** 1 Center for iPS Cell Research and Application (CiRA), Kyoto University, 53 Kawahara-cho, Shogoin, Sakyo-ku, Kyoto, 606-8507 Japan; 2 Department of Cell Modulation, Institute of Molecular Embryology and Genetics (iMEG), Kumamoto University, 2-2-1 Honjo, Tyuou-ku, Kumamoto, 860-0811 Japan; 3 Department of Pediatrics, Jichi Medical School, 3311-1 Yakushiji, Shimotsuke-shi, Tochigi 329-0498 Japan; 4 Department of Neurology, Graduate School of Medicine, Kyoto University, 54 Kawahara-cho, Shogoin, Sakyo-ku, Kyoto, 606-8507 Japan

## Erratum:

The original version of this article [1] unfortunately contained a mistake. The information in Table 1 was misrepresented.

In Table 1 in the information related to Alex2 clone, E63K should read E69K and in the information related to Alex3 clone, R276L (c.827G>T) should read L264P (c.791_792TG>CT). Additionally, the second column header has been modified from “clinical character” to “Diagnosis”.

An updated version of Table 1 has been provided below.

**Table 1:** Summary of iPSCs in this study

Clone name	Diagnosis	GFAP genotype	Sex	Age at onset	Age at sampling
HC1	healthy	wild	female	-	36
HC2	healthy	wild	female	-	67
HC3	healthy	wild	male	-	74
Alex1	Alexander disease type I	R239C (c.729C>T)	male	2	6
Alex2	Alexander disease type I	E69K (c.205G>A)	female	3	10
Alex3	Alexander disease type II	L264P (c.791_792TG>CT)	female	33	45

*Abbreviations*: *GFAP* Glial fibrillary acidic protein, *HC* Healthy control

Alex1 was generated from patient fibroblasts (GM16825) from Coriell Institute (Camden, NJ)
