# Supplementary figures and images for: Modeling Alexander disease with patient iPSCs reveals cellular and molecular pathology of astrocytes
Source: Acta Neuropathol Commun. 2016 Jul 11;4:69. doi: 10.1186/s40478-016-0337-0 (PMC4940830; doi:10.1186/s40478-016-0337-0)

# Supplemental Figure 1

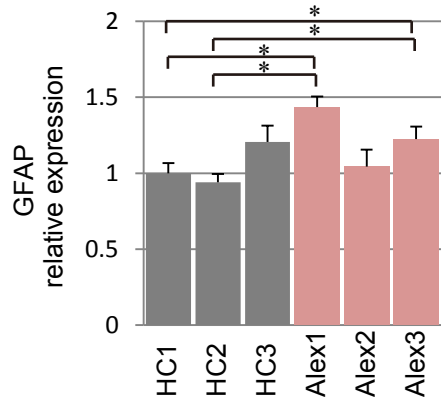

Supplement: Additional file 1: Figure S1. — GFAP expression of iPSC-derived astrocytes. Gene expression of GFAP was quantitatively analyzed with RT-qPCR. Two-way analysis of variance (ANOVA) showed significant variation. F (5, 12) =9.2490; p = 0.0008. Post hoc analysis revealed significant increases in GFAP expression in Alex1 and Alex3 (*, p < 0.05). Data represent mean ± SD (biological replicates, n = 3). (PDF 343 kb) [file 40478_2016_337_MOESM1_ESM.pdf]

# Supplemental Figure 2

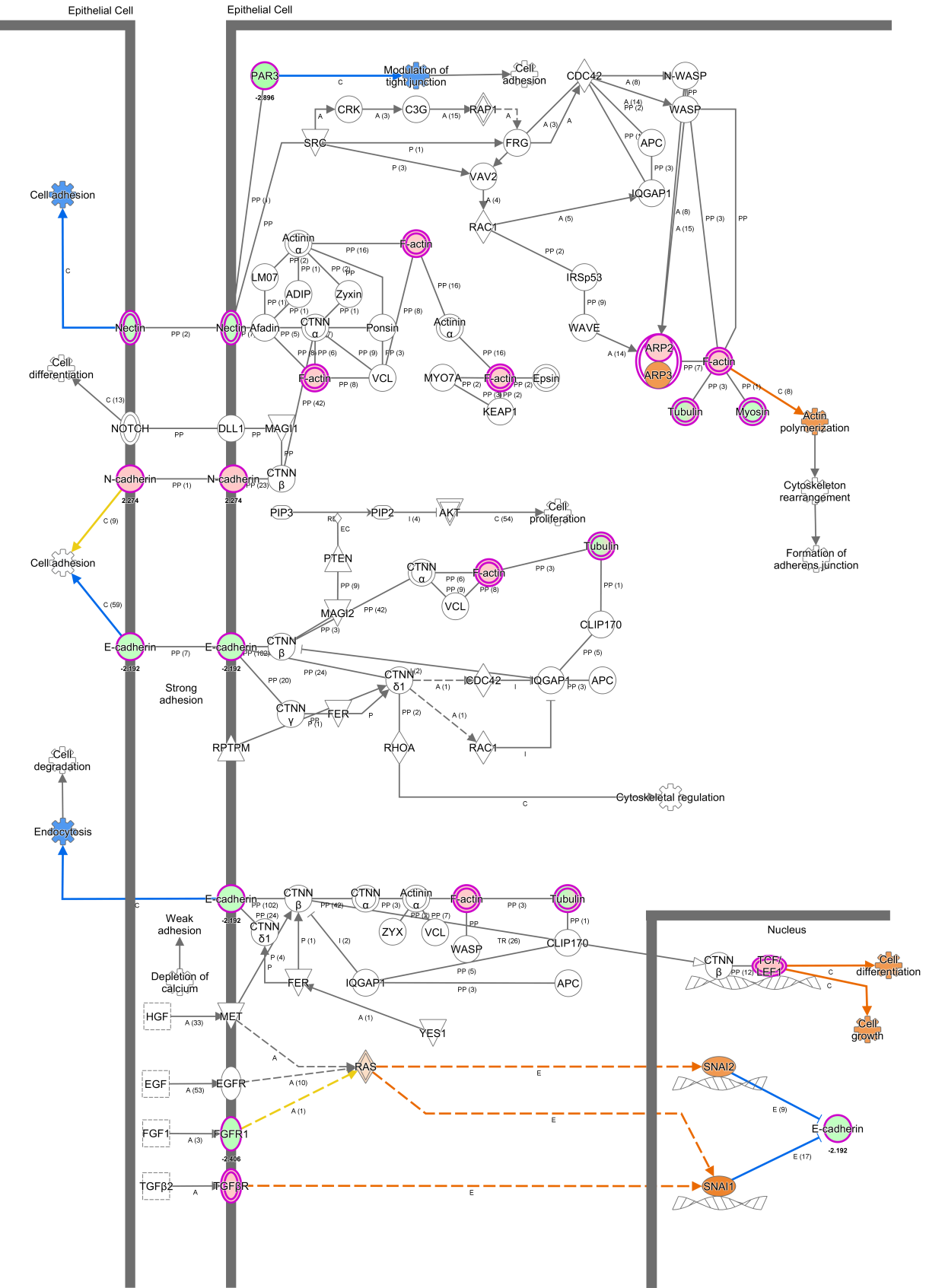

Supplement: Additional file 2: Figure S2. — Alteration in cellular adherence pathway. Gene expression changes were described within epithelial adherens junction signaling. Red to orange color = increased in Alexander disease. Blue to green color = decreased in Alexander disease, Grey color = not altered. (PDF 3.50 MB) [file 40478_2016_337_MOESM2_ESM.pdf]

# Supplemental Figure 3

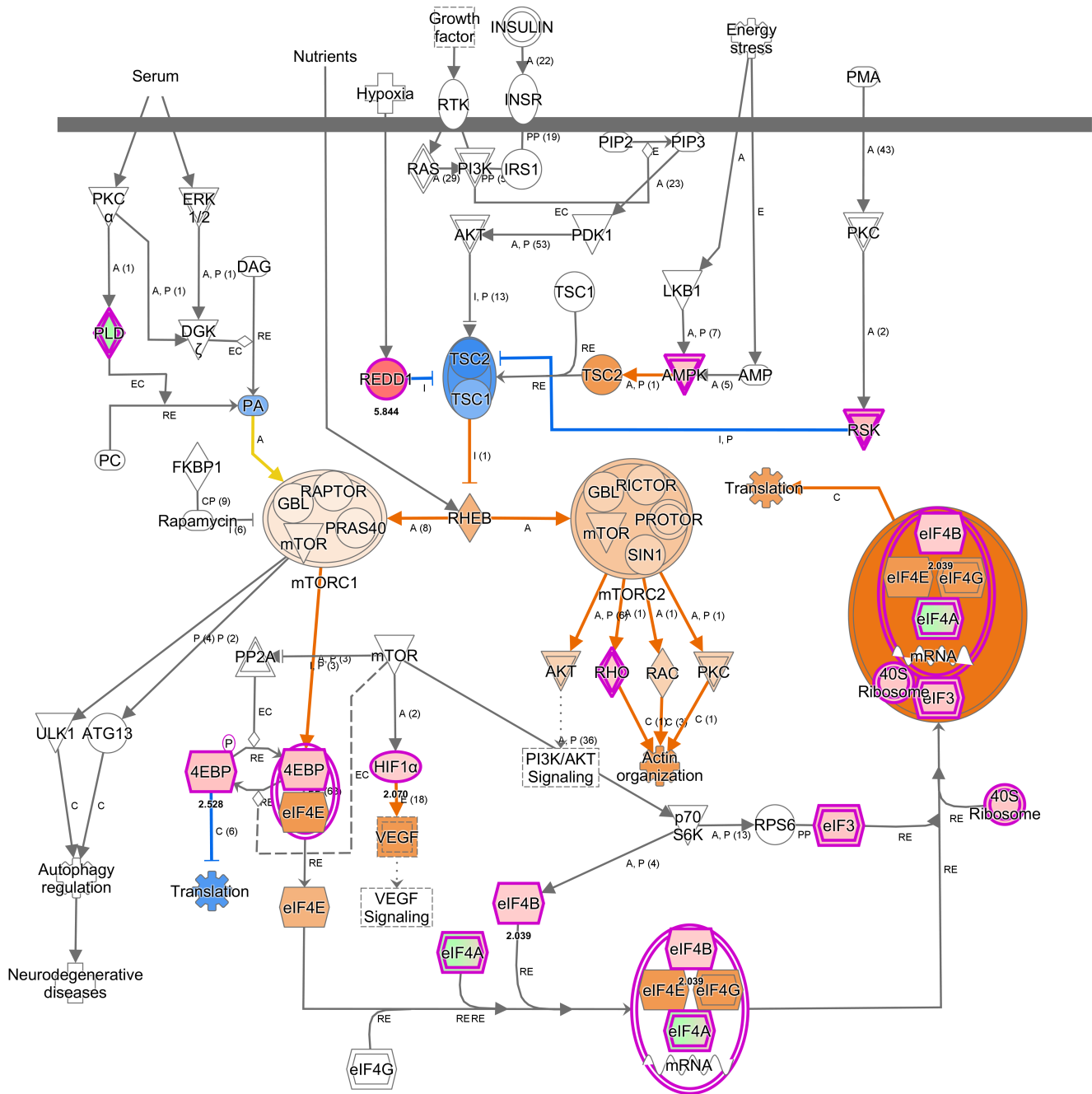

Supplement: Additional file 3: Figure S3. — Alteration in mTORC1/mTORC2 pathway. Gene expression changes were described within mTOR signaling. Red to orange color = increased in Alexander disease. Blue to green color = decreased in Alexander disease, Grey color = not altered. (PDF 3.63 MB) [file 40478_2016_337_MOESM3_ESM.pdf]

# Supplemental Figure 4

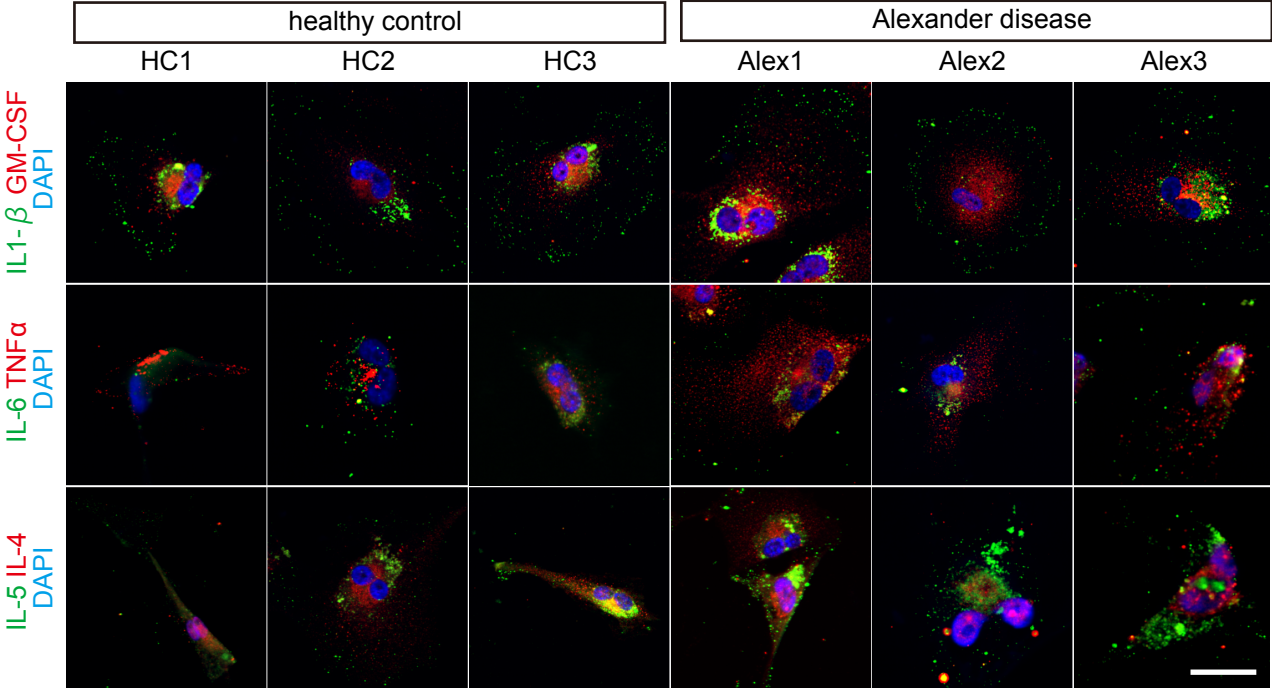

Supplement: Additional file 4: Figure S4. — Immunofluorescent study of cytokines in iPSC-derived astrocytes. iPSC-derived astrocytes showed positive staining of IL-1β, IL-6, IL-5 (green color) and , GM-CSF, TNFα, IL-4 (red color). Scale bar = 5 μm. (PDF 3.32 MB) [file 40478_2016_337_MOESM4_ESM.pdf]
